# Network-Based Asymmetry of the Human Auditory System

**DOI:** 10.1093/cercor/bhy101

**Published:** 2018-05-02

**Authors:** Bratislav Mišić, Richard F Betzel, Alessandra Griffa, Marcel A de Reus, Ye He, Xi-Nian Zuo, Martijn P van den Heuvel, Patric Hagmann, Olaf Sporns, Robert J Zatorre

**Affiliations:** 1 Montréal Neurological Institute, McGill University, Montreal, Quebec, Canada; 2 Department of Bioengineering, University of Pennsylvania, Philadelphia, PA, USA; 3 Signal Processing Laboratory 5 (LTS5), Ecole Polytechnique Fédérale de Lausanne (EPFL), Lausanne, Switzerland; 4 Department of Radiology, Centre Hospitalier Universitaire Vaudois (CHUV), Lausanne, Switzerland; 5 Brain Center Rudolf Magnus, UMC Utrecht, Utrecht, The Netherlands; 6 CAS Key Laboratory of Behavioral Science, Institute of Psychology, Beijing, People’s Republic of China; 7 Department of Psychological and Brain Sciences, Indiana University, Bloomington, IN, USA

**Keywords:** auditory, connectome, network, spreading

## Abstract

Converging evidence from activation, connectivity, and stimulation studies suggests that auditory brain networks are lateralized. Here we show that these findings can be at least partly explained by the asymmetric network embedding of the primary auditory cortices. Using diffusion-weighted imaging in 3 independent datasets, we investigate the propensity for left and right auditory cortex to communicate with other brain areas by quantifying the centrality of the auditory network across a spectrum of communication mechanisms, from shortest path communication to diffusive spreading. Across all datasets, we find that the right auditory cortex is better integrated in the connectome, facilitating more efficient communication with other areas, with much of the asymmetry driven by differences in communication pathways to the opposite hemisphere. Critically, the primacy of the right auditory cortex emerges only when communication is conceptualized as a diffusive process, taking advantage of more than just the topologically shortest paths in the network. Altogether, these results highlight how the network configuration and embedding of a particular region may contribute to its functional lateralization.

## Introduction

The brain is a complex network of anatomically connected and functionally interacting neuronal populations. These connectivity patterns span multiple spatial and topological scales (Lichtman and Denk 2011; Betzel and Bassett 2016), conferring the capacity for both specialized processing and multimodal integration among distributed systems. Increasing evidence suggests that the anatomical connectivity patterns may not be perfectly symmetric, however, with several systems marked by lateralized connection density and topological features (Iturria-Medina et al. 2011).

Auditory networks in particular display a pronounced tendency for functional asymmetry (Cammoun et al. 2015). Numerous studies have reported both structural and functional differences between the left and right auditory cortex, and have documented their differential contributions to a wide range of sensory and cognitive tasks, including speech (Giraud et al. 2007) and tonal processing (Zatorre and Gandour 2008). These asymmetries have also been observed at the network level, with asymmetric patterns of functional interactions or connectivity during specific tasks involving auditory processing, such as speech and language (Morillon et al. 2010) and pitch processing (Cha et al. 2014).

Recent evidence from stimulation studies raises the possibility that this lateralization is mediated by asymmetric anatomical connectivity and network embedding of the auditory cortices. For instance, stimulation of the auditory network by transcranial magnetic stimulation (TMS) elicits highly asymmetric patterns of activity and functional connectivity, with more widespread effects if the stimulus is applied over the right auditory cortex compared with the left (Andoh and Zatorre 2013). Importantly, individual differences in responses to stimulation are predicted by both interhemispheric anatomical connectivity and resting state functional connectivity (Andoh et al. 2015). Altogether, these studies suggest that the functional asymmetry of the auditory network may partly be a consequence of its topology, with the right auditory cortex better positioned to disseminate, exchange, and integrate neural signals with other systems.

Here we investigate whether the observed functional asymmetry of the auditory system can be attributed to the anatomical network embedding of left and right auditory cortex. Using connectivity patterns reconstructed from diffusion-weighted imaging (DWI) in 3 different datasets, we assess the propensity for left and right auditory cortex to maintain connections and potential communication pathways with the rest of the brain.

Importantly, we investigate a range of measures that embody different models of network communication. To assess the potential for left and right auditory cortex to communicate with the rest of the brain via shortest paths, we estimate the path length between these areas and the rest of the network (also referred to as closeness centrality or nodal efficiency). To assess the potential for these areas to communicate via an ensemble of paths, we estimate their communicability with the rest of the network (Crofts et al. 2011; Andreotti et al. 2014; Estrada and Hatano 2008; Crofts and Higham 2009; de Reus and van den Heuvel 2014). Finally, we use a simple spreading model in which focal perturbations in left and right auditory cortex develop into global signaling cascades that diffuse through the network (Granovetter 1978; Misic et al. 2015; Watts 2002). Unlike path length and communicability, the model is inherently dynamic, and allows us to trace the trajectories of putative signaling cascades. We hypothesize that if the lateralization of the auditory system has an anatomical origin, the network embedding of the primary auditory cortices will differ between the left and right hemispheres, with the right auditory cortex better positioned to communicate with, and influence, other areas.

## Materials and Methods

### Datasets

We performed all analyses in 3 DWI datasets. The main (discovery) dataset was collected at the Department of Radiology, University Hospital Center and University of Lausanne, (LAU; *N* = 40). We also included 2 replication cohorts, 1 from the Human Connectome Project (HCP; *N* = 215; (Van Essen et al. 2013)) and 1 from the Nathan Kline Institute Rockland Sample (NKI; *N* = 285; (Nooner et al. 2012)). Structural connectivity was reconstructed from DWI: diffusion spectrum imaging (DSI) for LAU, high-angular resolution diffusion imaging (HARDI) for HCP and diffusion tensor imaging (DTI) for NKI. Although dataset LAU had the fewest participants, we selected it as the main dataset to demonstrate our findings because of the quality of the DSI sequence. Below we describe the acquisition, processing, and connectome reconstruction procedure for each dataset in more detail.

#### LAU

A total of *N* = 40 healthy young adults (16 females, 25.3 ± 4.9 years old) were scanned at the Department of Radiology, University Hospital Center and University of Lausanne. Grey matter was parcellated according to the Desikan–Killiany atlas (Desikan et al. 2006). These regions of interest were further divided into 114 approximately equally sized nodes (Cammoun et al. 2012). Structural connectivity was estimated for individual participants using deterministic streamline tractography as implemented in the Connectome Mapping Toolkit (Cammoun et al. 2012), initiating 32 streamline propagations per diffusion direction for each white matter voxel. For more details regarding the acquisition protocol and reconstruction procedure see (Misic et al. 2015).

#### HCP

A total of *N* = 215 healthy young adults (112 females, 29.7 ± 3.4 years old) were scanned as part of the HCP Q3 release (Van Essen et al. 2013). Grey matter was parcellated according to the Desikan–Killiany atlas (Desikan et al. 2006). These regions of interest were further divided into 219 approximately equally sized nodes (Cammoun et al. 2012). Structural connectivity was estimated for individual participants using generalized q-sampling (GQI) (Yeh et al. 2010) and deterministic streamline tractography. For more details regarding the acquisition protocol and reconstruction procedure see (Misic et al. 2016).

#### NKI

A total of *N* = 285 healthy adults (112 females, 44.38 ± 19.7 years old) were scanned as part of the NKI initiative (Nooner et al. 2012). Grey matter was parcellated into 148 regions of interest according to the Destrieux atlas (Destrieux et al. 2010). Structural connectivity was estimated for individual participants using the Connectome Computation System (CCS) (http://lfcd.psych.ac.cn/ccs.html). For more details regarding the acquisition protocol and reconstruction procedure see (Betzel et al. 2016).

### Defining Auditory and Visual Regions

Primary auditory and visual cortex were delineated according to the Desikan–Killiany (for LAU and HCP) (Desikan et al. 2006) and Destrieux atlases (for NKI) (Destrieux et al. 2010). Both atlases are based on automated anatomical labeling of MR images using gyral and sulcal landmarks. Primary auditory cortex was defined as the “transverse temporal” (Desikan–Killiany) and the “G_temp_sup-G_T_transv” (Destrieux) nodes. Primary visual cortex was defined as the “pericalcarine” (Desikan–Killiany) and “S_calcarine” (Destrieux) nodes. None of these nodes were subdivided into smaller units than defined in the original atlases.

### Consensus Adjacency Matrices

Given recent reports of inconsistencies in reconstruction of individual participant connectomes (Thomas et al. 2014), as well as the sensitive dependence of network measures on false positives and false negatives (Zalesky et al. 2016), we adopted a group-consensus approach, whereby for each dataset we estimated edges that occur most consistently across participants (de Reus and van den Heuvel 2013; Roberts et al. 2016). In constructing a consensus adjacency matrix, we sought to preserve 1) the density and 2) the edge length distribution of the individual participants’ matrices (Misic et al. 2015; Betzel et al. 2016). The approach is conceptually similar to the procedures proposed by (de Reus and van den Heuvel 2013; Roberts et al. 2016).

We first collated the extant edges in the individual participant matrices and binned them according to length. The number of bins was determined heuristically, as the square root of the mean binary density across participants. The most frequently occurring edges were then selected for each bin. Thus, if the mean number of edges across participants in a particular bin is equal to *k*, we selected the *k* edges of that length that occur most frequently across participants. To ensure that interhemispheric edges are not under-represented, we carried out this procedure separately for interhemispheric and intrahemispheric edges. The binary densities for the final group matrices were 20.1% (LAU), 8.2% (HCP), and 11.1% (NKI) (Fig. S2).

### Communicability

Communicability (*C*_*ij*_) between 2 nodes *i* and *j* is a weighted sum of all paths and walks between those nodes (Estrada and Hatano 2008). For a binary adjacency matrix *A*, communicability is defined as follows:

$$C_{ij}=\overset{\infty }{\underset{n=0}{\sum }}\frac{{\left[A^{n}\right]}_{ij}}{n!}={\left[e^{A}\right]}_{ij}$$

with walks of *n* normalized by *n*!, such that shorter, more direct walks contribute more than longer walks.

### Linear Threshold Model

The linear threshold model (LTM) describes how a perturbation introduced at one or more seed nodes develops into a cascade and spreads through a network (Granovetter 1978; Watts 2002; Nematzadeh et al. 2014; Misic et al. 2015). The perturbation and subsequent cascade are modeled as an active state; any given node adopts this active state only if a certain threshold proportion of its neighbors have also adopted the active state. A form of contact percolation, the cascading behavior described by LTM has been extensively studied over a wide range of networks, including spatially embedded brain networks (Kaiser and Hilgetag 2010; O’Dea et al. 2013; Misic et al. 2015). The models capture how generic focal perturbations, such as the transduction of a sensory stimulus, spread through connected neuronal populations (see Discussion for a discussion of the neurobiological interpretation and limitations).

Formally, the state of a node *i* at time *t* is denoted as a binary variable $r_{i}\left(t\right)=\;\left\{0,1\right\}$, with only 2 possible states: active (1) or inactive (0). At initialization (*t* = 0), the entire network is inactive, except for a subset of activated seed nodes. The model is then updated synchronously at each time step according to the rule:

$$r_{i}\left(t+1\right)=\{\begin{array}{c} 1,\;\theta s_{i}<\underset{j\in N_{i}}{\sum }r_{j}\left(t\right)\\ 0,\;otherwise \end{array}$$

Thus, at each time step the state of node *i* depends on its neighborhood, *N*_*i*_ and specifically on the number of incident connections (degree or strength, *s*_*i*_). The node adopts the active state only if the proportion of inputs from active nodes exceeds the threshold *θ*. In the case of binary networks, the threshold represents the proportion of a node’s neighbors that must be active to propagate the cascade. The model can be naturally extended to weighted and directed networks, whereby the threshold represents the proportion of a node’s total weighted inputs (strength) that must be connected to active neighbors. In all scenarios, the fundamental performance measure is the adoption or spread time *A*_*ik*_, from seed node *i* to target node *k*. Spread times are a dimensionless model construct, conditioned on the size and density of the underlying graph. They do not correspond to physical time units in any straightforward manner.

How does the threshold influence spreading dynamics? At lower thresholds, nodes require fewer neighbors to be active at time *t* to become active at time *t *+ 1. Thus, nodes will be activated at the earliest possible time step, and the cascade will effectively propagate along the shortest path. As the threshold is increased beyond the inverse of the highest degree/strength in the network, cascades can no longer influence the most highly connected nodes and do not spread through the whole network (Fig. S3). Specifically, at higher thresholds it is more difficult to activate nodes, as more of their neighbors need to be active, so the dynamics are more dependent on local connectivity. At lower thresholds, the dynamics are less constrained by local connectivity and more influenced by global topology.

In the present study, we selected the threshold using the following criteria. The threshold had to be low enough to ensure that all perturbations will cause a complete cascade, so that spread times from the left and right auditory cortex could be unambiguously compared (Fig. S3). Increasing the threshold biases spreading away from shortest paths, with much of the spreading process occurring via alternative paths as well. As a result, spread times become less correlated with path length at greater thresholds (Fig. S4a). We therefore selected a threshold at which cascades could reach the whole network. In all 3 datasets, this corresponded to *θ =* 0.05.

How sensitive is the main effect of interest—the difference in spread time for perturbations originating in left and right auditory cortices—to this parameter setting? Figure S4b shows the effect of varying the threshold on the left–right auditory cortex asymmetry. At lower thresholds spreading is similar to shortest path routing, and there are no significant differences between left and right auditory cortex. As the threshold is increased, there is a range in parameter space ([0.04 0.09]) where spreading is significantly faster from the right auditory cortex compared with left auditory cortex.

## Results

White matter networks (connectomes) were reconstructed from DWI in 3 cohorts of healthy adults. We investigated the lateralization of primary auditory cortex by quantifying the topological distance from the left and right auditory cortex to the rest of the brain. We estimated topological distance using 3 measures, each of which makes different assumptions about the nature of inter-regional communication: path length (the minimum number of edges between 3 nodes), communicability (weighted sum of all walks between 2 nodes) and spread time (the time required for a signaling cascade to spread from one node to another; see Materials and Methods for details of model implementation). The spread time is a dynamic measure of inter-regional communication, estimated by simulating how a focal perturbation develops into a global signaling cascade and spreads through the network.

### Right Auditory Cortex is More Topologically Central

To assess the statistical reliability of differences in the anatomical centrality or embedding between the left and right auditory cortex, we used nonparametric tests. In the case of communicability, we used Wilcoxon signed-rank tests (Wilcoxon 1946). In the case of path length and spread times, which are not continuous, we used unpaired permutation tests (10 000 repetitions).

**Figure 1.**
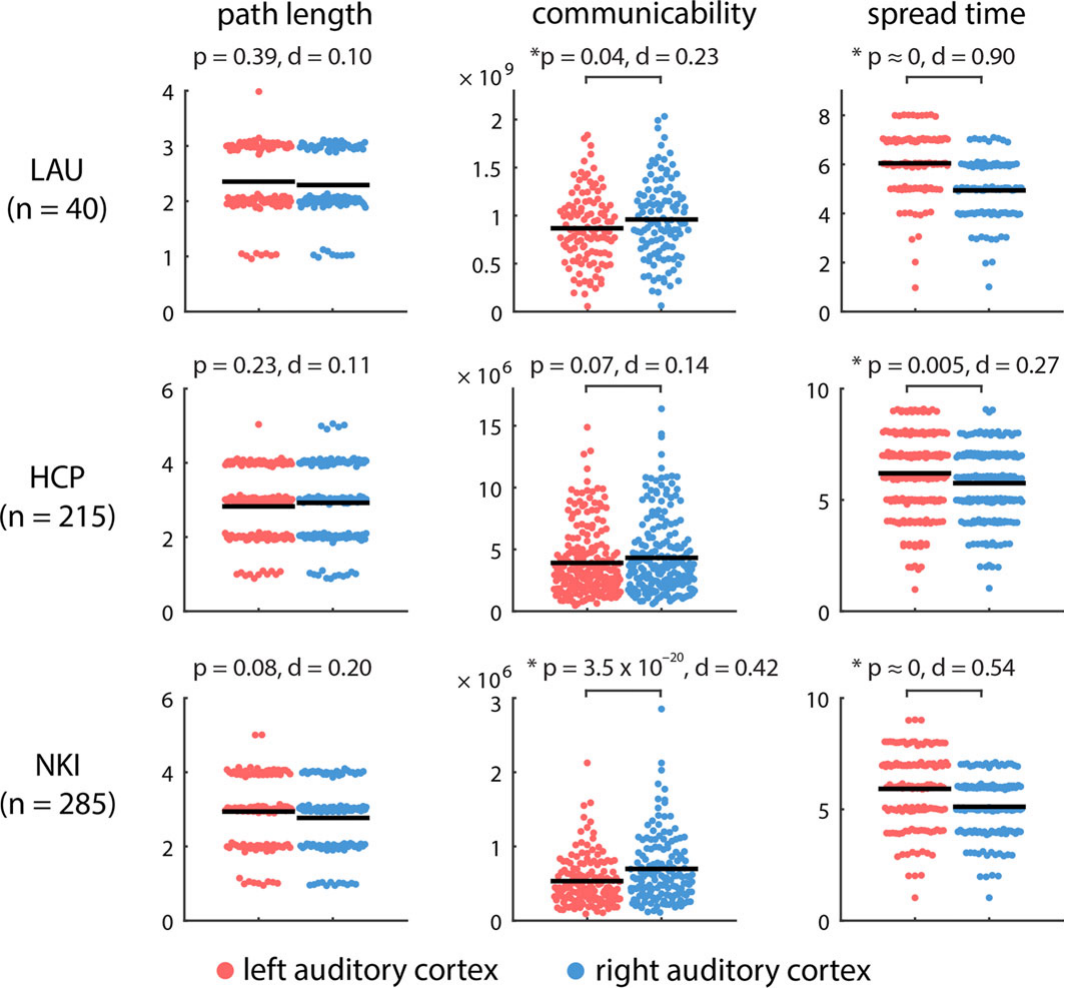
Communication distance from auditory cortices to the rest of the brain. The centrality of left and right auditory cortices was estimated by their topological distance to other brain areas in terms of path length, communicability and spread time. Shorter path length, greater communicability, and shorter spread times indicate greater proximity. Mean values for each distribution are indicated by solid horizontal black lines. For visualization, a random horizontal jitter was added to all points. In the case of path length and spread time, which are discrete-valued variables, an additional vertical jitter was added to all points.

Two salient findings emerge. First, there are differences in the anatomical embedding of left and right auditory cortex, but these differences only emerge when one considers communication metrics that assume diffusion of information, rather than shortest path routing. Namely, we find that the left and right auditory cortex are indistinguishable in terms of their path length to the rest of the brain (*P* = 0.39). Conversely, the right auditory cortex is topologically closer to other brain areas in terms of diffusive spreading, including greater communicability (*P* = 0.04) and faster spreading times (*P* < 10^−5^) (Fig. 1, top row). As shown in Figure S2, the 2 hemispheres are comparable in their connection density, so the observed effects are more likely to have arisen from differences in topology.

Second, despite significant differences in acquisition protocol, processing parameters, resolution and tractography algorithm, these results were replicated in the HCP and NKI datasets (Fig. 1, middle and bottom rows). In both datasets, left and right auditory cortex were statistically indistinguishable in terms of their path length to the rest of the network (*P* = 0.23 in HCP; *P* = 0.08 in NKI), while the spread time for cascades originating in right auditory cortex was significantly faster compared with those originating in left auditory cortex (*P* = 5 × 10^−3^ for HCP; *P* < 10^−5^ for NKI). The asymmetry was not only statistically significant, but also associated with a large overall effect size in all 3 datasets (Cohen’s *d* = 0.90, 0.27, 0.54 for LAU, HCP, and NKI datasets, respectively).

### Auditory Asymmetries are Cumulative

We next sought to pinpoint the origin of these anatomical asymmetries. Are left–right topological asymmetries due to a specific anatomical connection, or do they reflect a more global, cumulative effect? To answer this question, we used the spreading model because 1) it is dynamic, allowing us to trace the evolution of each signaling cascade through individual nodes and connections, and 2) the spread time measure consistently displayed the greatest effect size for the left–right asymmetry (Fig. 1).

**Figure 2.**
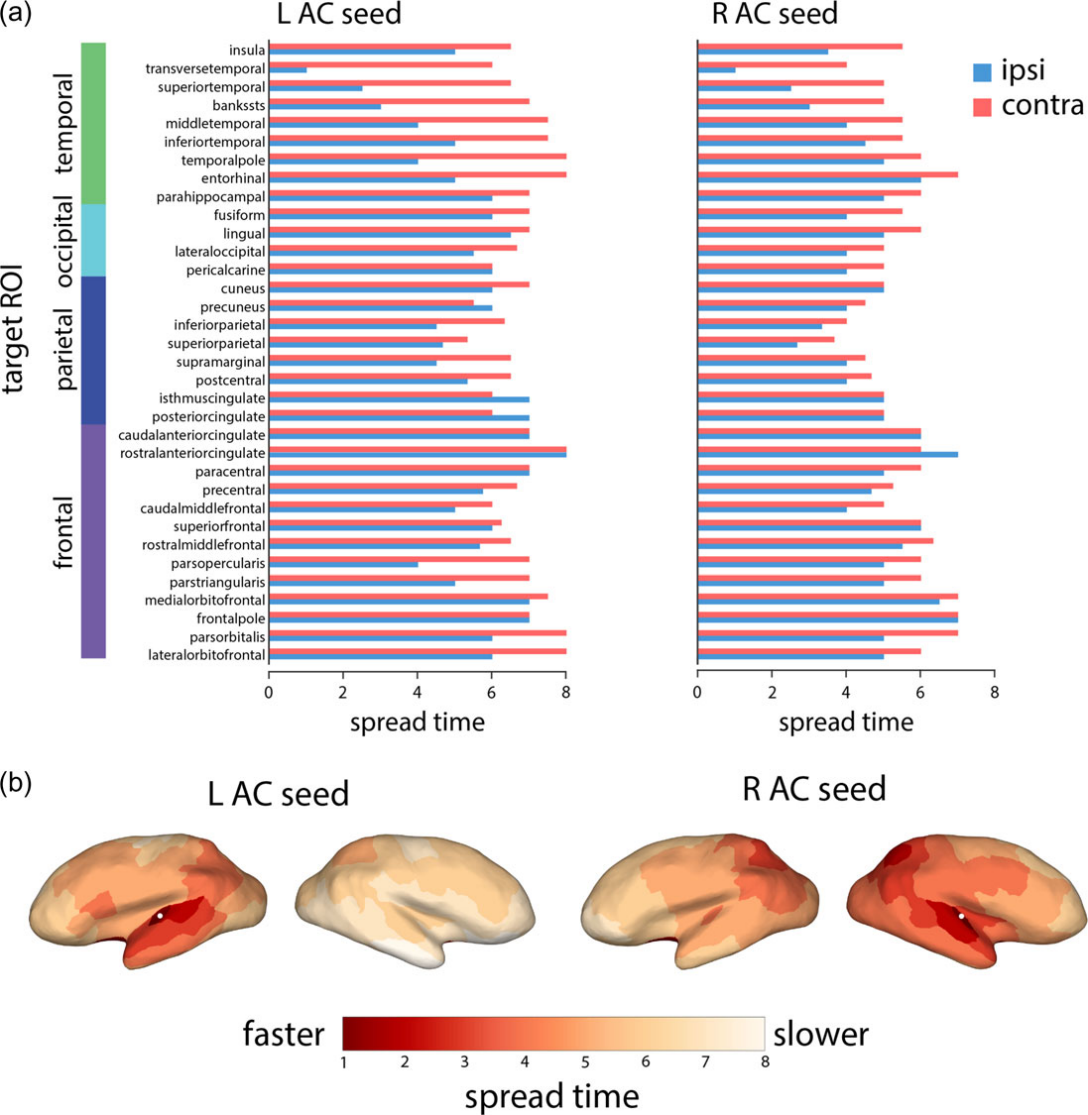
Simulated spreading from auditory cortices to specific target regions. (a) Spreading times to other nodes of the network, separated by lobe and hemisphere (blue for ipsilateral areas, orange for contralateral areas). (b) Spreading times for left and right auditory seeds projected to the cortical surface. The projected locations of the primary auditory nodes are indicated by white dots.

To investigate how the cascade spreading trajectories differed between the left and right auditory cortices, we further investigated spread times to specific targets. Figure 2 shows spread times for the left and right auditory seeds separately, stratifying the target ROIs into those contralateral and ipsilateral to the auditory seed node. We note 3 trends: 1) consistent with Fig. 1, spread times are generally faster from the right auditory seed, 2) spread times are faster for ipsilateral compared with contralateral targets (*P* = 5.19 × 10^−6^ and *P* = 1.35 × 10^−7^ for left and right auditory cortex, respectively), and 3) the biggest discrepancies between ipsilateral and contralateral targets are observed for temporal lobe targets, suggesting that much of the observed asymmetry is driven by less efficient communication between the left auditory cortex and the contralateral temporal lobe. Comparable results were observed in the 2 replication datasets, where signals originating from right auditory cortex reach the contralateral temporal lobe faster than signals originating from the left auditory cortex (Fig. S1).

### No Comparable Asymmetry in the Visual System

**Figure 3.**
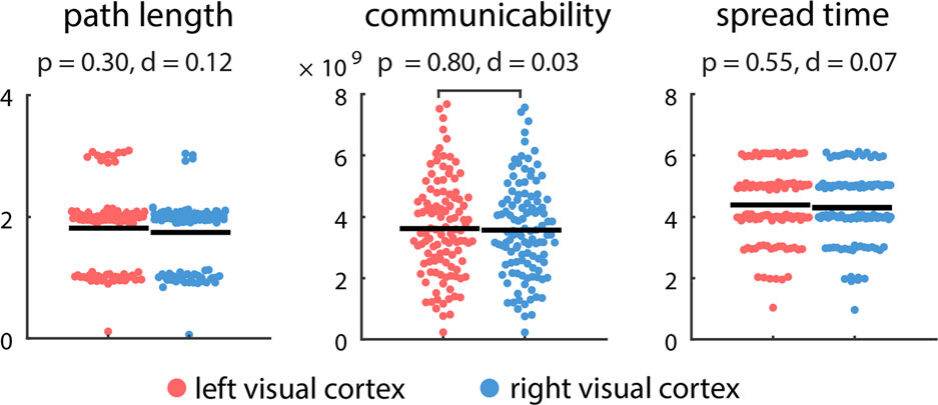
Communication distance from primary visual cortices to the rest of the brain. The centrality of left and right visual cortices was estimated by their topological distance to other brain areas in terms of path length, communicability and spread time. Shorter path length, greater communicability, and shorter spread times indicate greater proximity. Mean values for each distribution are indicated by solid horizontal black lines. For visualization, a random horizontal jitter was added to all points. In the case of path length and spread time, which are discrete-valued variables, an additional vertical jitter was added to all points.

While our results suggest a consistent asymmetry of the auditory cortices, it is possible that this lateralization is not unique to the auditory system, but perhaps a more general feature of sensory systems. To investigate this possibility, we repeated the analyses described above, but with a focus on primary visual (pericalcarine) cortex (Fig. 3). Unlike the auditory cortex, there was no evidence to suggest hemispheric asymmetry, with no statistically significant differences in path length (*P* = 0.30), communicability (*P* = 0.80), or spread time (*P* = 0.55).

## Discussion

The present investigation reveals a network-level asymmetry of the auditory system across multiple datasets. We highlight 3 principal findings: 1) the right auditory cortex is better integrated in the connectome, facilitating more efficient global communication, 2) these differences emerge only when communication processes are assumed to involve more than just the topologically shortest paths, and 3) much of the asymmetry is driven by differences in communication pathways to the opposite hemisphere.

### Lateralization of Auditory Networks

These findings support the notion that the functional asymmetry of the auditory system can be at least partly attributed to its embedding in the global anatomical network. Converging evidence from activation studies (Giraud et al. 2007; Zatorre and Gandour 2008), functional connectivity (Morillon et al. 2010; Cha et al. 2014), anatomical connectivity (Andoh et al. 2015; Cammoun et al. 2015) and stimulation (Andoh et al. 2015; Andoh and Zatorre 2013) points toward the possibility that the right auditory cortex is better positioned to communicate with and influence other systems. Our results suggest that the asymmetry could be at least partly explained by differences in several anatomical pathways, and that these differences potentially “accumulate” as information travels from auditory cortex towards more distant areas.

Specifically, our results suggest that the asymmetric influence of left and right auditory cortex is most pronounced with respect to the contralateral hemisphere. Spreading toward proximal areas in the ipsilateral hemisphere proceeded at a comparable pace for the 2 seeds, but the differences became more pronounced as the cascades coursed through the contralateral hemisphere, with the greatest differences observed for contralateral temporal lobe areas (Fig. 2). Building on previous reports that individual differences in the strength of auditory transcallosal pathways are related to TMS-induced modulation of interhemispheric functional connectivity (Andoh et al. 2015), our results point to the possibility that these asymmetries are also partly due to indirect pathways. Interestingly, a previous study of left–right asymmetries found that the connectivity patterns and lateralization of the auditory cortex may be more nuanced; while several anterior–posterior projections emanating from the auditory cortex were stronger on the right, other, mainly ventral–dorsal projections, were found to be stronger on the left (Cammoun et al. 2015). Altogether, these studies raise the possibility that differences in anatomical connectivity impart a distinct functional profile on the left and right auditory cortices.

While the present results emphasize the existence of a lateralized auditory network, it is important to note that this lateralization may be more general and may manifest in other systems as well (Corballis 2017). Although we found no evidence to suggest a similar lateralization in primary visual cortex, other studies have reported differences in anatomical connectivity for the 2 hemispheres. For instance, lateralization of connectivity and centrality is observed in several areas, with the right hemisphere displaying a more highly interconnected architecture, resulting in shorter average path lengths (Iturria-Medina et al. 2011). This observation suggests that the rightward asymmetry of the auditory system observed in the present study may be part of a broader pattern and warrants further investigation.

The present findings may be interpreted in the light of long-standing models of hemispheric specialization that have proposed various organizational principles to explain the phenomenon. Half a century ago, based on human lesion data, Semmes (1968) postulated that hemispheric functional asymmetries could be explained on the basis of more focal representation of function on the left compared with a more diffuse representation on the right. This idea and other related concepts have been debated over many years without resolution (Bradshaw 1981), one of the problems being the rather vague nature of the description, and the lack of clear neuroanatomical basis of it. This idea may now be reinterpreted in light of the asymmetric patterns of spread of activity described here: rather than reflecting more diffuse organization as such, the enhanced connectivity of the right auditory cortex with other parts of the brain may lead to greater integration of functional processes across widely distributed areas, which might manifest as a more diffuse pattern in response to lesions; conversely the more restricted connectivity of left auditory cortex would be associated with more specific interactions especially within the left hemisphere, leading to more focal lesion effects.

Note that the present macroscopic models only make predictions about the capacity for communication among relatively large neuronal populations. It is possible that local, microscopic connectivity patterns follow different distributions. For example, some authors have posited that enhanced temporal resolution in left auditory cortex may be related to enhanced myelination and enhanced transmission times in local circuits (Seldon 1981a, 1981b, 1982). The present findings are also compatible with a long-standing conjecture that hemispheric specialization may be related to interhemispheric conduction times (Ringo et al. 1994), so that computations that require relatively rapid interactions across regions may be better supported by local circuitry within a hemisphere. Speech processing, for instance, has been proposed to depend on critical intrahemispheric computations relating auditory, motor, and other structures within the left hemisphere (Morillon et al. 2010), and may be linked to enhanced auditory temporal resolution (Boemio et al. 2005; Zatorre et al. 2002). Such speech-related processes may thus benefit from the more focal left-side intrahemispheric organization we describe, because the fastest spread times from the left AC are found within a more local network, including not only adjacent regions in the left temporal neocortex, but also in the left inferior frontal gyrus, within Broca’s area, a network that is classically associated with auditory language functions. The possibility that this result reflects some relevant feature of a language-specific network is intriguing but requires further work. More generally, these findings highlight the need for multiscale models and measures that capture connectivity patterns across spatial scales.

A number of prior studies have also reported enhanced white-matter tracts within the left-hemisphere speech system compared with the right (Parker et al. 2005; Catani et al. 2007; Thiebaut de Schotten et al. 2011), in keeping with a more tightly organized intrahemispheric system. In addition, Iturria-Medina et al. (2011) point out that the right hemisphere show higher graph-theoretic indices of efficiency and interconnectivity than the left, again broadly consistent with our findings. Other, more local patterns of anatomical asymmetries within auditory cortices that have been described in the literature (Boemio et al. 2005; Hutsler and Galuske 2003; Marie et al. 2015; Meyer et al. 2014; Penhune et al. 1996; Schneider et al. 2005) can now also be re-examined in light of the long-range anatomical connectivity asymmetries described here.

Our anatomical findings also fit well with more recent reports of functional asymmetries. For example, Tomasi and Volkow (2012) report greater short and long-range connectivity in the right temporal cortex compared with the left, consistent with better transfer of information from right auditory-related areas to the rest of the brain. Liu et al. (2009) report greater left-hemisphere functional connectivity from resting-state date for several seed regions, including left superior temporal gyrus, indicating that this region has greater exchange of information with other left-hemisphere structures than its homolog on the right. Similarly, Gotts et al. (2013) report that resting-state connectivity patterns support greater within-hemisphere interactions on the left side (segregation) but greater between-hemisphere interactions on the right side (integration), a pattern that is once again consistent with our observations. Importantly, these authors demonstrated that the degree of integration vies segregation is related to individual differences in performance on cognitive task, thus demonstrating that degree of lateralization is related to behavioral ability (but see Catani et al. (2007)), and raising the possibility that cognitive function or dysfunction may in future be linked to the patterns of communication uncovered with the present methods.

### Beyond Shortest Path Communication

Interestingly, this auditory asymmetry is not observed when communication is assumed to occur exclusively along shortest paths, and only emerges when additional communication pathways are taken into account, as in the communicability and spread time measures. These results are part of a growing realization that distributed communication and synchronization in brain networks may proceed via alternative routes (Graham 2014; Fornito et al. 2016; Avena-Koenigsberger et al. 2018), with several recent methods developed to quantify path ensembles (Crofts and Higham 2009; Crofts et al. 2011; Andreotti et al. 2014; Avena-Koenigsberger et al. 2016; Grayson et al. 2016) and the potential for pathways to participate in the diffusion of information (Goni et al. 2014; Misic, Goni et al. 2014; Misic, Sporns et al. 2014). Other recent methodologies revolve around similar ideas, including controllability of linear time-shift invariant systems (Gu et al. 2015; Betzel et al. 2016), activity flow mapping (Cole et al. 2016), and simulated perturbations of ongoing oscillatory dynamics (Cocchi et al. 2016; Spiegler 2016). Our results highlight the need to consider the form of communication that a particular measure assumes and that measures founded exclusively on the concept of shortest paths may not adequately capture the richness and complexity of distributed computations in brain networks (Fornito et al. 2016).

### Methodological Considerations

Our findings of network-level asymmetry in spread of activation were replicated in 3 independent datasets with different diffusion imaging acquisition parameters. The replicability of the effect provides evidence that these asymmetries in the auditory system are quite robust. The consistency of the findings also suggests that the asymmetry is unlikely to be related to particular biases or artifacts inherent to any one diffusion acquisition or reconstruction model, since each data set uses different parameters. As further evidence of reproducibility, we note that in each of the 3 datasets we were able to observe similar right-sided asymmetric spreading advantages when the samples were split into male and female subgroups. There is a large and complex literature on possible interactions between sex and hemispheric asymmetry (Good et al. 2001; Tomasi and Volkow 2012); our findings are thus unlikely to be the last word on this topic, but at least at the level of the metrics used, and within the constraints of the available sample sizes, we can confirm that the principal asymmetric effect reported in the present study can be observed in both men and women.

Despite the replicability of the findings, it is important to note several limitations as well. First, our conclusions are based on networks reconstructed from DWI, a method known to be susceptible to false positives and negatives (Jones et al. 2013; Maier-Hein et al. 2017; Thomas et al. 2014). Inferring connectivity from local orientation fields is fundamentally an ill-posed problem, so without anatomical verification the present results must be interpreted with caution. Although we attempted to mitigate inaccuracies that may be present at the single-subject level by focusing on group-consensus networks derived from high-quality acquisitions in large samples of participants, and by repeating our analyses in multiple datasets, systematic errors or biases in the tractography procedure may still be present. In addition, networks derived from diffusion imaging are by definition undirected, limiting inferences about directionality of influence. These considerations highlight the need for new techniques for noninvasive mapping of white matter projections in the human brain. At the same time, the fact that we obtained similar results from data collected with different acquisition sequences, which are likely to have different biases towards false positives versus false negatives, indicates that the asymmetry is unlikely to arise merely from these factors.

Second, our ability to capture network asymmetries is contingent on the accuracy of our communication models. All network measures—including simple path length—assume some form of communication, but how information is transferred among topologically distant neural elements remains unknown. Although we estimated the centrality of the auditory network across a spectrum of communication mechanisms, from shortest path communication to diffusive spreading, it is nevertheless possible that inter-regional communication proceeds via a different mechanism.

## Conclusion

The present study highlights how the network configuration and embedding of a particular region may contribute to its functional lateralization. As our ability to image, reconstruct, and stimulate specific neural circuits advances, theoretical models of how perturbations and influence spread through brain networks will become increasingly important. These techniques will ultimately help to create a closer correspondence between structural and functional properties of specific areas and systems.

## Supplementary Material

Supplementary Data
