# Supplementary material for: Network-Based Asymmetry of the Human Auditory System
Source: Cereb Cortex. 2018 May 2;28(7):2655–64. doi: 10.1093/cercor/bhy101 (PMC5998951; doi:10.1093/cercor/bhy101)
Supplement: Supplementary Data [file bhy101_suppl1.zip › bhy101Misic_Suppl_Legends.docx]

**Figure S1 | Simulated spreading from auditory cortices to specific target regions in the (a) HCP and (b) NKI datasets.** Perturbations are introduced in the left and right auditory cortices (L and R AC). Spreading times to other nodes of the network are stratified by lobe and hemisphere (blue for ipsilateral areas, orange for contralateral areas).

**Figure S2 |** **Binary density of the group-level networks.** Density is shown for each group-consensus network, as well as for each of the subgraphs corresponding to the hemispheres.

**Figure S3 | The effect of threshold on global spreading.** The linear threshold model (LTM) was used to simulate the effects of focal perturbations at each of the 114 nodes in the Lausanne (LAU) dataset. At low thresholds, the whole network can be trivially activated. As the threshold is increased, some nodes become increasingly difficult to activate, and the total proportion of the network that is activated begins to decrease.

**Figure S4 | Choosing a threshold.** (a) The effect of threshold on the correlation between spread time and path length. When the threshold is low, the dynamics of the model resemble a breadth-first search and spread times are perfectly correlated with path length. As the threshold is increased, spreading is driven away from shortest paths (as evidenced by the decreasing correlation) and more akin to diffusion. (b) The effect of threshold on left-right auditory cortex (AC) asymmetry. The difference in spread time between left (L) and right (R) auditory cortex is shown as a function of threshold. The difference is quantified as a Cohen's d effect size. At lower thresholds, when spreading is similar to shortest path routing, there are no significant differences between L and R AC. As the threshold is increased, there is a range in parameter space where spreading is significantly faster from the right auditory cortex compared to left auditory cortex (Wilcoxon rank sum test, FDR corrected).
