# Supplementary figures and images for: Network-Based Asymmetry of the Human Auditory System
Source: Cereb Cortex. 2018 May 2;28(7):2655–64. doi: 10.1093/cercor/bhy101 (PMC5998951; doi:10.1093/cercor/bhy101)

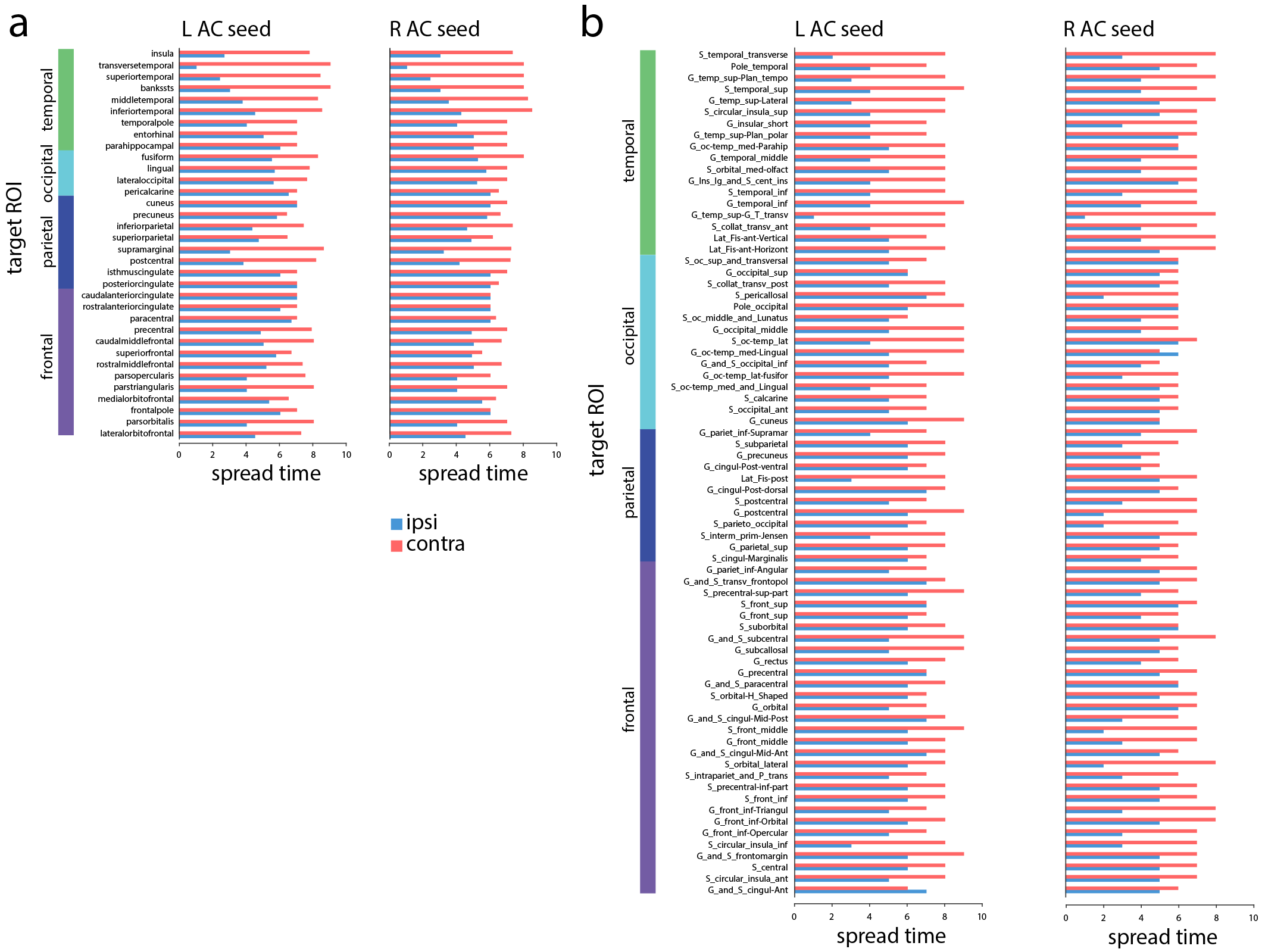

Supplement: Supplementary Data [file bhy101_suppl1.zip › bhy101Misic_Fig_S1.tif]

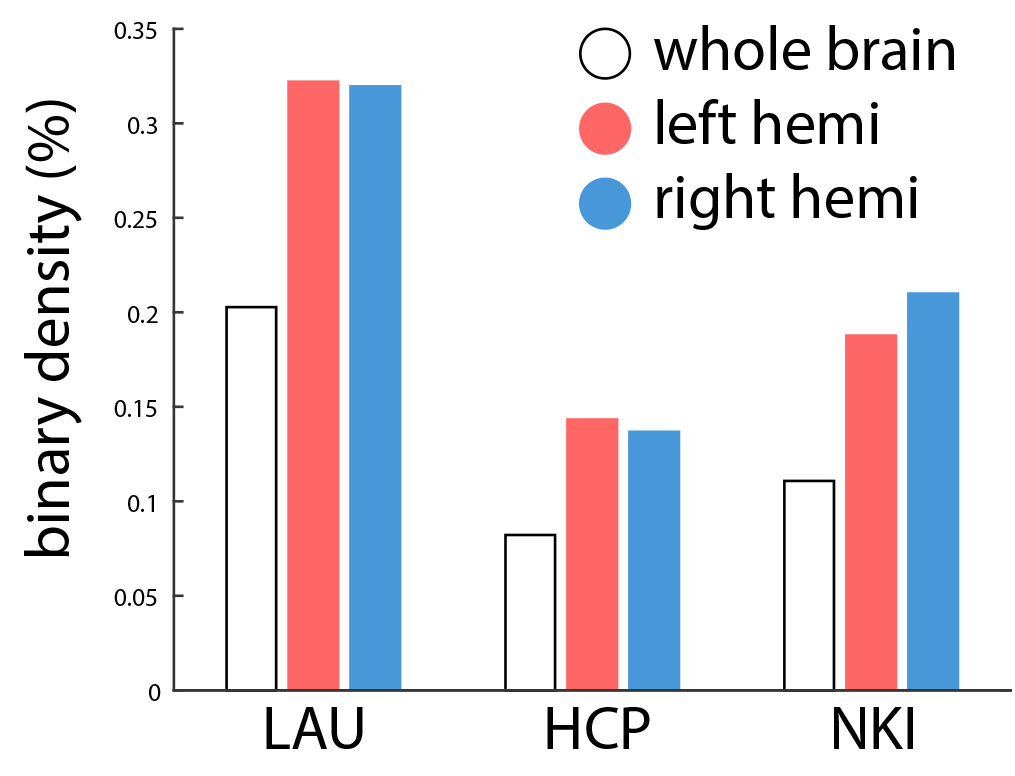

Supplement: Supplementary Data [file bhy101_suppl1.zip › bhy101Misic_Fig_S2.tif]

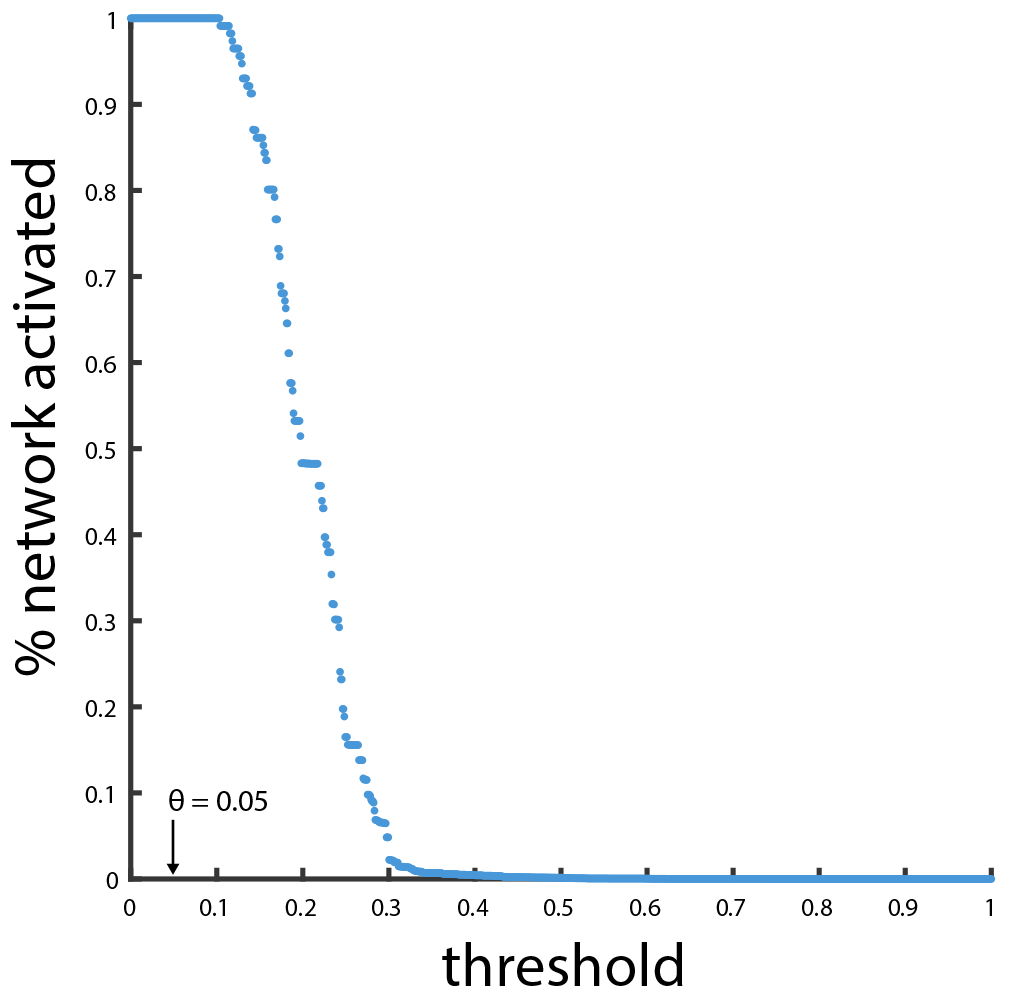

Supplement: Supplementary Data [file bhy101_suppl1.zip › bhy101Misic_Fig_S3.tif]

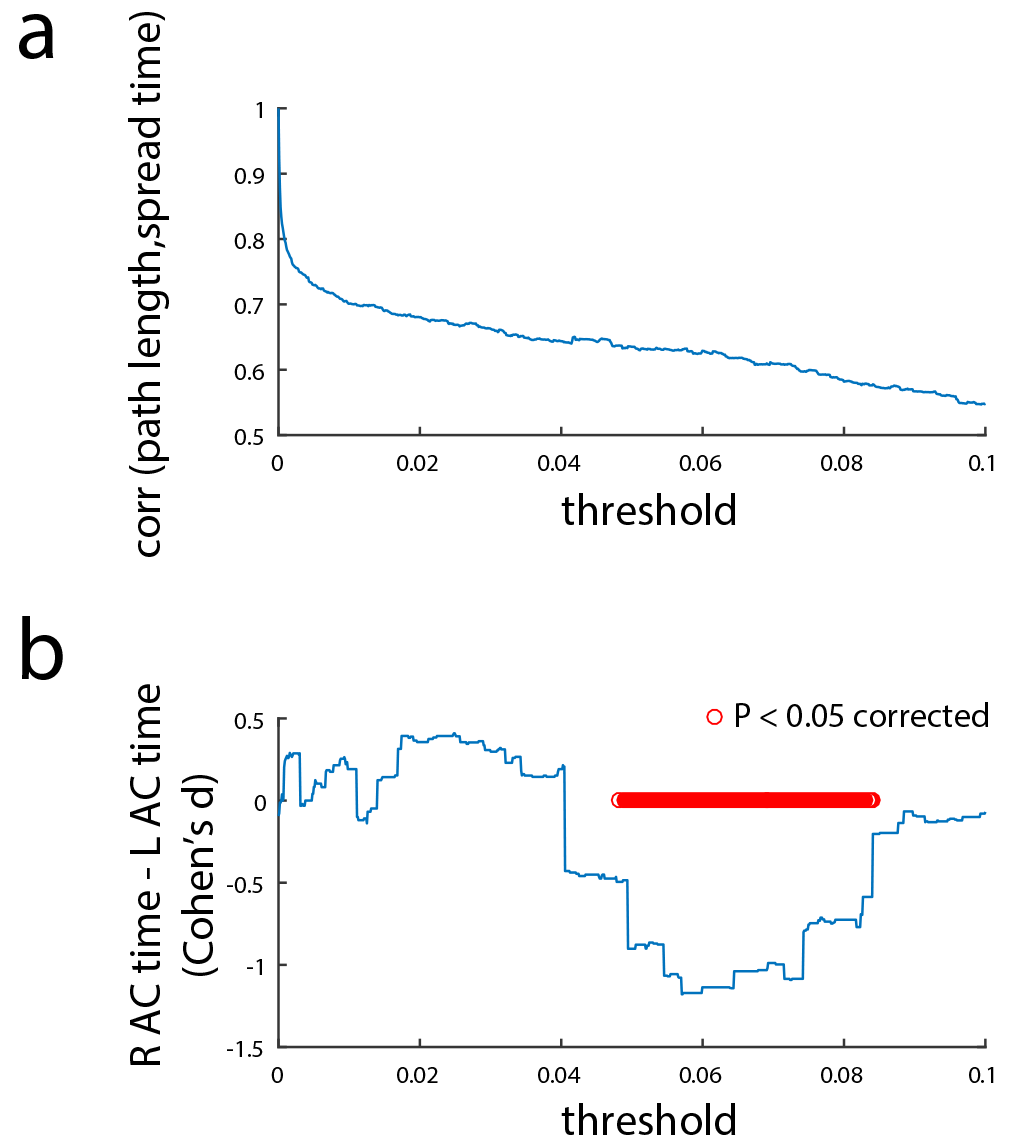

Supplement: Supplementary Data [file bhy101_suppl1.zip › bhy101Misic_Fig_S4.tif]
